# Pollution Breaks Down the Genetic Architecture of Life History Traits in *Caenorhabditis elegans*

**DOI:** 10.1371/journal.pone.0116214

**Published:** 2015-02-25

**Authors:** Morgan Dutilleul, Benoit Goussen, Jean-Marc Bonzom, Simon Galas, Denis Réale

**Affiliations:** 1 Département des Sciences Biologiques, Université du Québec À Montréal, Montréal, Canada; 2 Institut de Radioprotection et de Sûreté Nucléaire (IRSN), PRP-ENV/SERIS/LECO, Cadarache, Bât 183, BP 3,13115 St Paul-lez-Durance, France; 3 Université de Montpellier 1, Faculté de pharmacie, Laboratoire de Toxicologie, BP 14491, F-34093 Montpellier Cedex 5, France; 4 Unit “Models for ecotoxicology and toxicology” (METO) INERIS Parc ALATA, BP2 60550 Verneuil-en-Halatte, France; Fred Hutchinson Cancer Research Center, UNITED STATES

## Abstract

When pollution occurs in an environment, populations present suffer numerous negative and immediate effects on their life history traits. Their evolutionary potential to live in a highly stressful environment will depend on the selection pressure strengths and on the genetic structure, the trait heritability, and the genetic correlations between them. If expression of this structure changes in a stressful environment, it becomes necessary to quantify these changes to estimate the evolutionary potential of the population in this new environment. We studied the genetic structure for survival, fecundity, and early and late growth in isogenic lines of a *Caenorhabditis elegans* population subject to three different environments: a control environment, an environment polluted with uranium, and a high salt concentration environment. We found a heritability decrease in the polluted environments for fecundity and early growth, two traits that were the most heritable in the control environment. The genetic structure of the traits was particularly affected in the uranium polluted environment, probably due to generally low heritability in this environment. This could prevent selection from acting on traits despite the strong selection pressures exerted on them. Moreover, phenotypic traits were more strongly affected in the salt than in the uranium environment and the heritabilities were also lower in the latter environment. Consequently the decrease in heritability was not proportional to the population fitness reduction in the polluted environments. Our results suggest that pollution can alter the genetic structure of a *C. elegans* population, and thus modify its evolutionary potential.

## Introduction

Anthropogenic activities can disturb biological functions. In particular, environmental pollution has negative consequences on an organism’s life history and fitness, and on the demography of the population. However, individuals may not be affected in the same way because these reactions depend on their individual genotypes and phenotypes [1,2]. Pollution can thus create differential survival and reproductive successes, which in turn can lead to microevolution in populations [3,4]. The evolutionary potential of life history traits depends on the strength of the selection pressures acting on each of these traits in the population, their level of additive genetic variation, and the magnitude and sign of their genetic associations [5–7]. The two latter are included in the matrix of additive genetic variance and covariance (**G**) [8]. The **G** matrix reflects the evolutionary constraints on the independent evolution of traits imposed by pleiotropy or linkage disequilibrium. An estimation of the **G** matrix is thus a necessary step towards the estimation of a population’s evolutionary potential [6,9].

Evolutionary change in quantitative traits can be described by the multivariate extension of the breeder’s equation [7,8]. This equation is only applicable when the **G** matrix is stable over time and across environmental conditions. Some theoretical and empirical investigations have revealed a strong **G** matrix stability over time [10], despite the effects of factors, such as climate change [11]. However, other studies demonstrated that the stability of the **G** matrix can be altered [12–14], even following short-term environmental changes (i.e. from the first generation of the environmental change) [15,16]. Moreover, changes in environmental conditions may not only affect the additive genetic and environmental variance of traits [17,18], but also their covariance (reviewed by [19]). These studies contradict the hypothesis of a stable **G** matrix, even over short time periods. Furthermore, under harsh environmental conditions, a decrease in the genetic variance of traits may reduce the adaptive potential of populations, despite strong selection pressures occurring in this type of environment, and may thus prevent microevolutionary changes [20,21]. Therefore, using **G** matrix information on one particular set of environmental conditions to predict the evolutionary potential of a population subjected to rapid environmental changes may be questionable. Re-estimating the **G** matrix for the new environmental conditions may be the only way to estimate a population’s evolutionary potential. This is particularly important for studies that predict the evolutionary consequences of novel and fast environmental changes caused by anthropogenic effects, such as the effect of acute pollution [22]. In this paper, we use a quantitative genetic approach to test the hypothesis that the **G** matrix for life history traits remains stable in a population of *Caenorhabditis elegans* subject to different pollutants.

We chose to work on the effects of two pollutants: uranium (uranyl nitrate) and a high salt (NaCl) concentration. They affect *C*. *elegans* in different ways. High salt exposure is an extreme hypertonic stress that provokes a rapid loss of water and solute content in cells. *C*. *elegans* attempts to regulate this loss by synthesising glycerol through transcriptional upregulation of an enzyme (*gpdh-1*) in the intestine and hypodermis, which are both in direct contact with the external medium [23,24]. However several other genes, such as genes regulating heat shock, and cytoskeletal and trehalose biosynthesis, also regulate hypertonic stress [25]. Uranium is a heavy metal that has higher chemotoxic than radiotoxic effects because of its low specific activity [26]. Uranium severely affects the intestinal epithelium in the earthworm, *Eisenia fetida* [27]. The presence of uranium in the environment increases *C*. *elegans’* expression of metallothionein-1 (*mtl-1*), which interferes with U-accumulation in cells; probably by sequestering and removing uranium from the cells [28].

*C*. *elegans* is often used as a model when studying quantitative genetic parameters because of its short life cycle and ease of handling [29]. For example, the genetic correlation structure (i.e. the genetic correlation between traits in an environment) of life history traits estimated from recombinant inbred lines differed between high and low food densities [30] and low and high temperatures [31] when they were at levels that were known to stress *C*. *elegans*.

This study used the isofemale line method [32] to analyse the changes in the genetic structure of the life history traits of a *C*. *elegans* population in a control environment and in environments polluted with either uranium or a high salt concentration. Life history traits are directly involved in the demographic response of a population to any rapid environmental change and they generally encompass many other traits that can affect both survival and reproduction. Working on life history traits gives us a broad picture of the evolutionary response of an organism to changing environmental conditions. Furthermore, in the absence of detailed information on the tolerance mechanisms used by *C*. *elegans* when subject to salt and uranium stress, life history traits can be used to examine what type of response this organism may show when exposed to these new environmental conditions. Our hypotheses were (i) that a genetic structure (i.e. heritability and genetic correlations) exists between traits in the three different environments; (ii) that the expression of this structure can be altered in the polluted environments compared to the control environment because of the drastic changes in environmental conditions; and (iii) that genetic correlations exist for the same trait across the three different environments.

## Material and Methods

*C*. *elegans* experiments does not require approval as specified by general guidelines of the CNRS (France) regarding experimentation using invertebrates

### Isofemale line technique

In this study, we applied the isofemale line technique (ILT), which is a method that is commonly used on wild populations *of Drosophila sp*. (review by [32]). The relationship between narrow-sense heritability (*h* ^2^) and the coefficient of intraclass correlation (*t*) provided by ILT is not very obvious. Nevertheless, *t* does give a better heritability estimate than the half-sib design or the parent–offspring regression method. For example, the technique provides measures for about 95 ± 15% of the morphometric trait heritabilities in natural populations of *D*. *melanogaster* [32], thus yielding a close estimation of additive genetic variance. It is a useful tool for rapidly estimating genetic variability and the genetic correlation between traits, and can also compare these genetic parameters between different traits and different environments (or even different populations). Furthermore, ILT can be easily applied to *C*. *elegans* because of its androdioecious breeding system (i.e. self-fertilization of hermaphrodites and facultative outcross with males). Indeed, after mating, each isogenic line can be conserved as an inbred line. The term “heritability” has been used throughout this paper instead of “the coefficient of intraclass correlation”.

### Nematode culturing

We used a stock population of *C*. *elegans*, composed of a mixture of 16 wild isolates [33], to obtain a large genetic diversity. The population, containing more than 30% males, was maintained under laboratory conditions for 140 generations, where recombination–selection equilibrium was mostly achieved without significant loss of genetic diversity [33]. We put 500 individuals in a 9 cm diameter Petri dish containing NGM-modified (i.e. nematode growth medium) agar and HEPES buffer [34]. We produced six replicated experimental populations. The plates were seeded with 1 ml of a 20:1 mixture of *Escherichia coli* strain OP50 (OD_600nm_ = 3) as the food source. Every 3 days we washed the nematodes (about 20,000 individuals) off the plates with an M9-modified solution (containing HEPES buffer) and kept each replicate in 15 ml Falcon tubes. The number of individuals in a tube was estimated using five sample drops of 5 µl, following Teotónio *et al*. [33]. Then a volume corresponding to 500 individuals, for all developmental stages, was placed in a fresh Petri dish. The nematodes were cultured throughout the experiment at 20°C and 80% RH.

### Creation of lines

After repeating this protocol about 40 times (~40 generations despite the discrete overlapping of developmental stages), we selected 100 gravid hermaphrodites and placed them on a new Petri dish. This was repeated for all six replicates. Potential evolutionary changes caused by the changing environmental conditions should be similar in both the treatment and control environments. We allowed the hermaphrodites to lay eggs for a 2 h period, and then transferred approximately 200 eggs to a new Petri dish (200 eggs per replicate). After 40 h, when sexual differentiation was visually possible, but individuals were not yet mature (i.e. L3 larval stage [35]), we randomly picked a single hermaphrodite and a single male from the different Petri dishes and placed them separately (by couples) in six-well tissue-plates. We created 14 couples.

The NGM in the wells was seeded with 150 µl of a 5:1 mixture of OP50. After leaving the plates in a laminar flow hood for 1 h to allow them to dry, the plates were then top-exposed to UV doses for 90 s to stop bacterial growth (Bio-Link Crosslinker; λ = 254 nm; intensity = 200 µwatt.cm^-2^). The main aim of this UV treatment was to avoid differential bacterial growth in the control and polluted plates. Reproduction by mating produces a high frequency of males in the progeny, whereas hermaphrodite self-fertilization produces only 0.1% males [36–38]. Therefore, 72 h after the separation of the couple, we checked to see if the ratio of males and hermaphrodites in the progeny was approximately 1:1, to guarantee that mating between the hermaphrodite and the male was successful. We then picked one L3 stage hermaphrodite in each of the progeny of 14 couples to initiate 14 isogenic lines. This was done 72 h after couple creation to avoid self-fertilization, which is often a risk in the first few hours after mating [39]. Each line was reproduced by self-fertilization for two generations in a 6 cm Petri dish, in the same environment as used above.

### Contamination design and measurement of traits

When the second self-fertilization generation had appeared, we transferred 20 gravid hermaphrodites of each line to a new Petri dish. This was considered as t = 0. After 2 h of egg-laying, the eggs were individually placed in a 12-well tissue culture plate. Twelve eggs per line and per environment were placed in three different NGM media: control, uranium (addition of 1.1 mM U [uranyl nitrate: UO_2_(NO_3_)_2_·6H_2_O; Sigma–Aldrich, France]), and high salt concentration (addition of 308 mM NaCl). The wells were seeded with 75 µl of a 5:1 mixture of OP50 (same UV treatment as the six-well plates). In our experiment, the term “isogenic” does not correspond exactly to our lines, as there was heterozygosity in our population [33]. However, the individuals had been reproduced twice by self-fertilization before the experiment; so we expected a significant reduction in heterozygosity [40–42].

Individuals growing in salt showed a slower development rate than in the control individuals and those in the uranium. Therefore, individuals were transferred twice into a new well at 96 h in the control and uranium environment, or at 168 h in the salt environment and then again 36 h later. Hatched progeny were counted the day following each transfer to measure brood size, which is an index of fecundity. We photographed the individuals using a stereomicroscope (Olympus SZX12, 1.6 x 90 magnification) with a computer-connected camera (Nikon D5000). Body length was measured at 72 and 144 h using a micrometer scale measure and ImageJ software [43]. These two measurement times corresponded approximately to the population’s onset and to the end of the egg-laying period in the control environment, respectively. These two measures were called early growth and late growth in our study. Survival was estimated by counting the number of parental individuals still alive at 144 h.

### Genetic parameter estimations

We estimated the genetic parameters by using a Bayesian model approach in the MCMCglmm package for the generalized linear mixed-effects model [44] in the R software [45]. We used the multivariate generalized linear mixed-effects models for different traits within the same environment (a quadrivariate model per environment) and for the same trait across different environments (a trivariate model per trait). The models did not contain fixed effects, but we included lines as random effects to estimate the between and within-line variance for each trait. We modelled survival using a binary error structure and the Gaussian model for the other traits. Expanding the binomial data into binary data allowed us to fit a model that could estimate the correlation between survival and the other traits. Estimations were generated using a Bayesian approach so that we could obtain the entire posterior distributions of (co)variance matrices of traits rather than only point estimates. To avoid any biased results caused by the fact that trait mean values differed, we rescaled the traits prior to analysis by subtracting each value by the mean of the sample and dividing it by twice the standard deviation [46]. After several priors had been tested, we obtained a proper prior [*nu* = *k* – 1 + 0.002] with a very low variance parameter [*V* = diag(*k*) * *V*_*p*_ * 0.05], where *V*_*p*_ is the phenotypic variance and *k* is the dimension of *V* (i.e. number of traits). There was one exception for survival. In this case we fixed the environmental (also called the residual) variance to one. For the multivariate analyses with four different traits, we used the models to estimate different random and environmental variances and covariances between the pairs of traits. In the models that analysed the same trait across the three different environments, the traits were measured on different individuals, which fixed the environmental covariance to zero (see S1 Table for comparisons between the models, with or without genetic covariance, between traits allowed in the prior). Despite the slightly informative priors used in the models, starting with a low variance and any kind of covariance allowed us to say that the generated (co)variances were tangible. After verifying the convergence of the parameter values (i.e. number of iterations, burn-in phase and thinning), and autocorrelation issues, we retained 2 500 000 iterations with an initial burn-in phase of 500 000, from 1000 samples per analysis [44].

We estimated heritability in the multivariate models for traits within environments using *t* = *nVb* / (*nVb* + (*n* – 1)*Vw*), where *Vb* and *Vw* were respectively the between and within-line variance and *n* was the number of lines [32]. Following David *et al*. [32], we also estimated genetic correlations without correcting for variance and covariance within-line, although *n* was less than 20. Using this correction generated the posterior distribution for correlations containing values greater than 1. We used the posterior mode of the distribution (i.e. the value that appears most often in the obtained distribution) to estimate the heritability, and genetic and phenotypic correlations as quantitative genetic parameters. For each trait, we also estimated the phenotypic [*V*_*P*_ = *Vw* + *Vb*], environmental [*V*_*E*_ = *V*_*P*_ *—V*_*G*_] and genetic variances [*V*_*G*_ = *tV*_*P*_], and the genetic covariance between traits using ${\text{Cov}}_{1,2}=r_{\text{G}}\sqrt{V_{G1}V_{G2}}$, where *r*_G_ corresponds to genetic correlation between traits 1 and 2; and *V*_G1_ and *V*_G2_ are the genetic variance for trait 1 and 2, respectively (see S1 Fig.). We assumed that each estimate was significantly different from zero when its 95% Bayesian highest posterior density intervals (HPDIs) were not zero. We also tested the differences between an individual’s traits and the genetic parameters in different environments. We assumed differences to be significant between two environments when 95% HPDIs produced by the subtraction between the posterior distributions of the trait in the two polluted environments did not include zero.

## Results

The phenotypic values for all traits were lower in the salt environment than in the uranium and the control environments (95% HPDIs after subtraction did not include zero; Table 1). When compared to the control environment, fecundity decreased by 55.0% in the uranium environment and by 86.4% in the salt environment (95% HPDIs after subtraction did not include zero). The same pattern was found for both early and late growths. Survival in the uranium environment was similar to that of the control environment (95% HPDI after subtraction did not include zero), but it nearly halved in the salt environment.

**Table 1:** Average trait values of 14 C. *elegans* isogenic lines subjected to three different environments.

	Mean ± sd			Percentage
Environnement	Fecundity	Early growth	Late growth	Survival at 144h
Control	173.8 ± 43.5 a	953.1 ± 131.3 a	345.7 ± 106.9 a	80.9 ^a^
Uranium	78.2 ± 26.4 ^b^	721.5 ± 108.8 ^b^	184.2 ± 100.1 ^b^	88.8 ^a^
Salt	23.6 ± 34.1 ^c^	223.2 ± 79.6 ^c^	127.2 ± 121.0 ^c^	46.6 ^b^

Fecundity is measured by the number of eggs produced by a hermaphrodite. Early and late growth is recorded as the increase in total length (µm) between 0 and 72 h, and from 72 to 144 h. A difference is significant when the 95% intervals of highest posterior density of subtraction between the posterior distributions of a trait in two environmentsdid not include zero. They are represented by different superscript letters.

**Fig 1:**
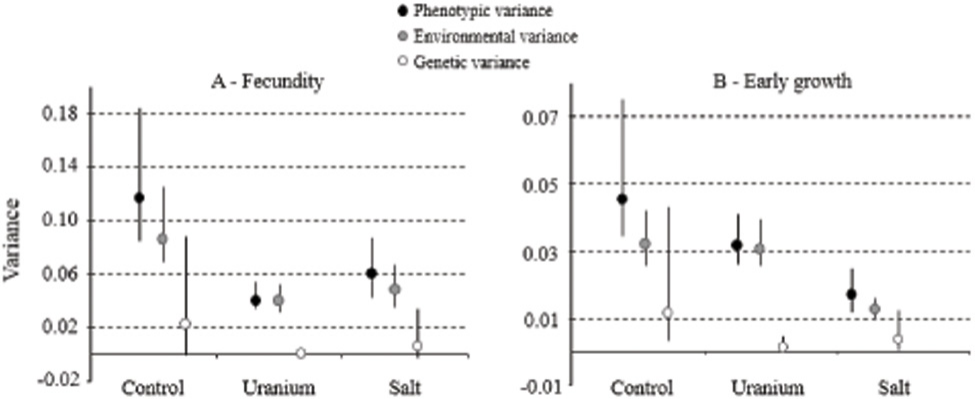
**Trait variance estimates for** *C*. *elegans* **in the different environments.** Variances are presented with their 95% intervals of Bayesian credibility. (A) fecundity and (B) early growth. Phenotypic variance (*V*_*P*_) is split into environmental (*V*_*E*_) and genetic variances (*V*_*G*_). Estimates were obtained using multivariate models for different traits within the same environment.

Heritability estimates varied between 0.07 and 0.31 in the control environment and were only significantly higher than zero for fecundity and early growth (Table 2). In the uranium environment, we found lower (i.e. 0.04 to 0.08) and non-significant heritabilities (Table 3). In the salt environment, heritability values varied between 0.07 and 0.27, but were only significant for early growth (Table 4). Heritability did not differ significantly between the control and the salt environments for fecundity and early growth, but heritability in the control environment was significantly higher than in the uranium environment (95% HPDIs after subtraction did not include zero). Phenotypic variance for these two traits declined in both the polluted environments compared to the control. However, in the uranium environment there was a larger reduction in genetic variance than in environmental variance, compared to the control (95% HPDIs after subtraction did not include zero, see Fig. 1 for fecundity and early growth and see S2 Fig. for information on late growth), which was directly related to lower heritabilities in this polluted environment (Table 2 and Table 3). Phenotypic variance for fecundity was twice as high in the control environment (*V*_*p*_ = 0.114 [0.083; 0.180]) than in the salt environment (*V*_*p*_ = 0.059 [0.042; 0.086]) and three times higher than in the uranium environment (*V*_*p*_ = 0.040 [0.034; 0.054] (see Fig. 1A and S3 Fig. for representations of phenotypic correlation structures). The difference was less pronounced for early growth but was still significantly different (Fig. 1B).

**Table 2:** Matrices for heritabilities, and phenotypic and genetic correlations for the traits when measured in the control environment.

CONTROL	Fertility	Early growth	Late growth	Survival
Fertility	***0.249 [0.036; 0.525]***	**0.904 [0.361; 0.965]**	**-0.912 [-0.987; -0.450]**	0.924 [-0.520; 0.985]
Early growth	**0.543 [0.345; 0.718]**	***0.314 [0.105; 0.608]***	**-0.780 [-0.967; -0.188]**	0.930 [-0.279; 0.991]
Late growth	-0.154 [-0.427; 0.093]	**-0.299 [-0.566; -0.101]**	*0.110 [-0.009; 0.424]*	-0.839 [-0.978; 0.588]
Survival	**0.695 [0.454; 0.881]**	0.346 [-0.007; 0.673]	0.132 [-0.310; 0.553]	*0.073 [-0.087; 0.394]*

Heritabilities (diagonal in italics), phenotypic (below the diagonal), and genetic correlations (above the diagonal) are presented along with their 95% Bayesian credibility intervals (in brackets). Values in bold are significant estimates.

**Table 3:** Matrices for heritabilities, and phenotypic and genetic correlations for the traits when measured in the uranium environment.

URANIUM	Fertility	Early growth	Late growth	Survival
Fertility	*0.039 [-0.075; 0.068]*	-0.023 [-0.635; 0.693]	-0.063 [-0.683; 0.784]	0.371 [-0.691; 0.863]
Early growth	**0.607 [0.485; 0.699]**	*0.042 [-0.073; 0.122]*	-0.521 [-0.835; 0.637]	-0.401 [-0.869; 0.717]
Late growth	0.140 [-0.031; 0.326]	**-0.347 [-0.479; -0.125]**	*0.034 [-0.059; 0.241]*	-0.720 [-0.948; 0.749]
Survival	**0.736 [0.411; 0.852]**	**0.512 [0.062; 0.724]**	0.208 [-0.372; 0.576]	*0.083 [-0.089; 0.154]*

Heritabilities (diagonal in italics), phenotypic (below the diagonal), and genetic correlations (above the diagonal) are presented along with their 95% Bayesian credibility intervals (in brackets). Values in bold are significant estimates.

**Table 4:** Matrices for heritabilities, and phenotypic and genetic correlations for the traits when measured in the salt environment.

SALT	Fertility	Early growth	Late growth	Survival
Fertility	*0.186 [-0.034; 0.442]*	**0.896 [0.514; 0.987]**	**0.774 [0.045; 0.966]**	0.892 [-0.804; 0.961]
Early growth	**0.551 [0.392; 0.723]**	***0.270 [0.001; 0.512]***	**0.811 [0.188; 0.967]**	0.901 [-0.825; 0.973]
Late growth	**0.537 [0.340; 0.694]**	**0.708 [0.584; 0.813]**	*0.126 [-0.039; 0.363]*	0.901 [-0.722; 0.967]
Survival	**0.404 [0.037; 0.742]**	**0.515 [0.144; 0.738]**	**0.725 [0.448; 0.880]**	*0.067 [-0.086; 0.217]*

Heritabilities (diagonal in italics), phenotypic (below the diagonal), and genetic correlations (above the diagonal) are presented along with their 95% Bayesian credibility intervals (in brackets). Values in bold are significant estimates.

We found moderate phenotypic correlations between traits in the three different environments (Table 2, Table 3 and Table 4). In all the environments, individuals that had a higher fecundity also showed faster early growth and had greater survival rates. The phenotypic correlation between early and late growths was negative in the control and in the uranium environment, but positive in the salt environment. In the control environment, fecundity and early growth were positively genetically correlated, but both of these traits were negatively correlated with late growth. The range of these correlations was strong (> 0.75 in absolute value terms, Table 2). Moreover, the genetic correlation between fecundity and late growth was hidden because phenotypic correlation was non-significant. The environmental correlation (*r*_e_) was opposite in sign to the phenotypic correlation, but non-significant (*r*_e_ = 0.100 [–0.128; 0.300], see S2 Table for environmental correlations). We did not detect any genetic correlation in the uranium environment. In particular, posterior modes for genetic correlations between fecundity and growth traits were close to zero (–0.023 and—0.063, Table 3). We found a strong genetic correlation between the same combination of traits in the salt and control environments (Table 2 and Table 4), but there was a change in sign for the correlation involving late growth due to the change in the genetic covariance signs (see S1 Fig. for genetic covariances between traits). We did not find any significant genetic correlations for survival in any of the environments (Table 2, Table 3 and Table 4). The environmental correlations were significant (S2 Table) for phenotypic correlations that had not been detected at the genetic level.

We found a positive and significant genetic correlation between the control and the uranium environment for fecundity (Table 5), which indicated a genetic dependence across environments. We also found a positive correlation for late growth across these two environments and a negative one for early growth between the two polluted environments, despite a slight zero overlap in the 95% HPDIs. We have not presented cross-environment genetic correlations for survival because of a potential lack of power.

**Table 5:** Cross-environment genetic trait correlations.

	Fecundity	Early growth	Late growth	Survival
Control-Uranium	0.718 [0.024; 0.968]	0.438 [-0.478; 0.838]	0.833 [-0.049; 0.954]	0.978 [-0.967; 0.998]
Control-Salt	0.444 [-0.585; 0.787]	0.087 [-0.473; 0.716]	0.554 [-0.488; 0.900]	0.967 [-0.940; 0.996]
Uranium-Salt	-0.070 [-0.746; 0.721]	-0.811 [-0.943; 0.076]	0.625 [-0.292; 0.942]	-0.943 [-0.999; 0.929]

Genetic correlations are presented along with their 95% Bayesian credibility intervals (in brackets).

## Discussion

Previous theoretical and empirical investigations have revealed differences in the genetic structure of morphological and life history traits between stressed and controlled environments [6,17,18]. This study has shown that living in a polluted environment can decrease both genetic and environmental variances. However, the greater reductions in genetic variance, compared to environmental variance, explained the reductions in heritability. Fecundity and early growth heritabilities were lower in the uranium environment than in the salt environment, despite the stronger effects of the salt environment on phenotypic traits. We found no genetic correlations in the uranium environment. There were strong positive genetic correlations between fecundity, early, and late growth in the control and salt environments. The correlations were positive, except the correlations involving late growth in the control environment. Cross-environment data suggested that there was a positive genetic correlation between the control and uranium environments for fecundity. There was a negative genetic correlation for early growth between the uranium and salt environments.

The heritability (*t*) results did not only yield additive genetic variance, probably because there is a large number of epistatic interactions due to an experimental bottleneck that was linked to how the lines were created. This additional effect would be cryptic in a large panmictic population. Other quantitative genetic designs may not be subject to this drawback. For example, an ‘animal model’ [47], using complete information on a particular pedigree, can provide very good estimates of quantitative genetic parameters. However, it is almost impossible to build up a pedigree for *C*. *elegans* because we cannot differentiate between individuals without separating them, as in our experiment. For the same reasons, and due to the difficulties in controlling all the offspring if they come from outcrossing or self-fertilization, it is very hard to create a half-sib/full-sib design experiment for this species. Besides, ILT has been shown to provide a better estimation of heritability than the parent–offspring regression method [48]. Therefore, ILT remains a very appropriate and convenient approach for quantitative genetic analyses of this species.

### Effects on heritability

Survival heritability was absent or extremely low (thus difficult to detect) in the control environment, compared to the other traits. This may be due to its genetic regulation. Survival is regulated by a few genes that have large effects contrary to growth or reproductive traits, which are regulated by many genes that have small effects on heritability [1,49] (in *C*. *elegans* [50]). Heritabilities in the control environment were moderate for early growth and fecundity, and low for late growth. Gutteling *et al*. [31] studied broad-sense heritability in recombinant inbred lines of *C*. *elegans* for body mass at maturation and fecundity at different temperatures. Heritability was similar to our study’s results for fecundity, but was higher for growth. However the variations can be explained by the differences in temperature between our study (20°C) and theirs (12°C and 24°C), which affects average growth, reproduction [51], and heritability [31].

The decrease in early growth and fecundity heritabilities in both polluted environments was consistent with several previous laboratory or field studies, although some studies showed the opposite response (reviewed by [17,18]). Heritability reduction could be caused by a decrease in additive genetic variance or an increase in environmental variance. Our results showed that both environmental and genetic variances were higher in the control, resulting in a phenotypic variance decrease in both polluted environments (Fig. 1). Despite the decrease in phenotypic variance, the reduction in heritabilities was mainly due to the reduction in (additive) genetic variance. Although a reduction in heritability is commonly caused by an increase in the environmental variance component, there can be cases where it results from a reduction in the genetic variance components [17]. In this latter case, the decrease in heritability could be triggered by a limitation in the genetic potential due to constraining developmental conditions [21], e.g. despite strong additive genetic effects responsible for individual differences in body size, the potential size may not be attained because of stress-induced reductions in the growth rate [52].

Heritabilities were extremely low in the uranium environment. Several studies have suggested that phenotypic variation in stressful environments seems to be related to the type of new environment being experienced [53,54]. The original populations, from which the wild isolates used in our study were taken, may have previously experienced various stresses that had affected the same sets of genes as the uranium affected. Moreover, the evolutionary response of a trait to selection pressure cannot occur in an environment where the trait heritability is zero, no matter how strong the selection pressures are. [21] showed a decrease in maternal genetic variance in a stressful environment for wild sheep, although the selection strength was strongly and positively correlated to the quality of the environment. Cadmium induced a broad-sense heritability reduction in *Daphnia magna* at 20°C, but apparently this was mostly due to the decrease in dominance variance [55]. Nonetheless, Hendrickx *et al*. [56] observed a strong decrease in growth heritability in the wolf spider, *Pirata piraticus*, and they associated this to a decrease in additive genetic variance caused by the cadmium environment. They suggested that the expression of growth in that species involved different sets of genes in different environments. In a stressful environment, gene expression governing normal traits can be masked by other alleles involved in detoxification [1]. Indeed, for several heavy metals, including uranium, tolerance is due to one or a few major genes in *C*. *elegans* [57,58], which could mask the expression of genes for quantitative traits, such as life history traits. Nonetheless, if heritability is only hidden, but is still existent, selection may still act on it. More studies are required to confirm the effects of polluted environments on heritability reduction.

### Cross-environment genetic correlations

We detected cross-environment genetic correlations between the control and uranium environments for fecundity and for late growth. This indicated a partial overlap between the genes involved in the expression of these traits in both environments [5], despite different genetic variances between the control and uranium samples. For the other measures, the 95% HPDIs largely overlapped zero, and therefore the correlation between the control and the polluted environments was probably negligible or the result may have been caused by a potential lack of power due to the number of lines in our experiment. Given that our aim was only to detect the presence of genetic correlations, rather than obtain an accurate measure of it, our method, based on the line mean, was applicable as it generally produced lower correlations than other methods, thus resulting in more conservative estimates (see [59]). All the posterior correlations with the control environment were either positive or close to zero, which implied that there was no trade-off for the measured traits between the uranium or salt environments and the control environment. Therefore, adaptation to uranium or to salt does not seem to be associated with a strong co-evolution between environments for these traits.

There was a negative genetic correlation for early growth between the uranium and salt environments, which indicated that there was a cost to adapting to the presence of these pollutants. Lopes *et al*. [60] found that once *C*. *elegans* had adapted to an environment polluted with a pesticide, there was no adaptation cost for reproduction when they were subsequently moved to the control environment or an environment polluted with a second pesticide. In contrast, Jansen *et al*. [61] suggested that there was an adaptation cost for *D*. *magna* populations that were exposed to a pesticide, and that it was conditional on the new environmental conditions. Adapted populations of *D*. *magna* suffered no costs in the control environment, but were more parasitized, which suggested that this population had a higher susceptibility to other stresses. In contrast, adaptation costs to cadmium were seen in the control environment, *D*. *magna* growth [62], and *D*. *melanogaster* fecundity [63]. The advantage of cross-environment estimates of genetic correlation, when measuring the potential adaptation cost to pollutants, is that the assessments can be performed over a short period of time compared to other approaches, such as experimental evolution. The absence or presence of an adaptation cost to pollutants revealed by this method can be confirmed by direct multigenerational experiments investigating adaptation to uranium and salt.

### Effects on genetic correlation structure

The strong genetic correlation for the control environment indicated a co-evolution between growth and fecundity (Table 2). Genotypes that grew faster before sexual maturity (early growth) were more fertile, but had a reduced late growth. This evolutionary strategy is generally shown by small, short-lived organisms, such as *C*. *elegans* (r-selection [6,64]). *C*. *elegans* produces juveniles as soon as possible [65]. Sperm production takes place during the larval stages and then stops at the molting stage, which produces the adults. The remaining germ line cells generate exclusively oocytes until the end of the egg-laying period [66]. Investment in growth during the costly oocyte production period directly affected fecundity [67]. Consequently, the likely outbreeding depression caused by hybridization between diverse wild isolates [68], to create the populations used in our experiments, may be the cause of its low fecundity (about 174 larvae in the control population, Table 1) compared to other populations of *C*. *elegans* [65]. However, outbreeding depression may have happened when the wild isolates were crossed and did not break down the genetic structure of the study population [33].

There were a number of genetic correlations for the populations raised in the salt environment and they covered the same combination of traits. When compared to the control, the changes in sign for correlations involving late growth in salt could be explained by the considerable developmental delay in this environment. Unlike the other environments, most individuals started laying eggs after 72 h, and even after 144 h. We could therefore say that early and late growth in the control and uranium environments corresponded to early growth in the salt environment and that the genetic structure between growth and fecundity was close to that of the control environment. In contrast, the genetic correlations in the control became zero in the uranium environment, especially the values for fecundity. Interestingly, in the uranium environment, the phenotypic correlations were similar to the control environment because there were significant environmental correlations (see S2 Table). Previous research has shown that the environmental correlations can have opposite signs to the genetic correlations, particularly when considering the acquisition and allocation of resources for both traits [6,69]. This could also explain the presence of highly significant environmental correlations, in the two polluted environments, where individuals have probably suffered from resources acquisition and allocation effects [25,27,28], compared to the control. This highlights the importance of looking at the genetic structure, even though the phenotypic structure might be similar in different environments. The genetic structure may have broken down, which would lead to a different evolution of life history traits [6,70–72].

Tolerance mechanisms that are governed by gene expression ensure the survival of individuals under highly stressful conditions, such as pollution, but they also affect the expression of genetic structure [1]. Shirley and Sibly [63] analysed the processes by which *D*. *melanogaster* increased its tolerance to cadmium. They suggested that the links between genes involved in fecundity, survival, and development period in a control environment may be disrupted due to the addition of tolerance genes that are expressed in the presence of cadmium. It is also the case for the natural birch-feeding insect, *Priophorus pallipes*. The genetic correlation between body size and development time was positive when foliage quality was good and negative or low when the resource quality was close to zero [73]. Evolutionary trajectories depend on the effects of environmental conditions on organisms [19]. Hence, even if selection experiments in polluted environments mean that we need to consider the temporal heterogeneity of the **G** matrix, e.g. the emergence of trade-offs [12,74,75], this study showed that the genetic structure had changed when individuals were subject to sudden pollution. In contrast, a change in the genetic structure in the uranium environment by selection on heterozygosity, for example, is hardly possible given that mortality was similar in the uranium and the control environments. There were also different degrees of change in the uranium and salt environments, which was probably dependent on their mode of action. Consequently, the evolution of the **G** matrix was already affected from the moment pollutants appeared in the environment. This is why it is important to investigate why some pollutants or specific genetic conditions promote **G** matrix stability or instability.

### Relationship between heritability and fitness

The decrease in heritability was higher in the uranium environment than in the salt environment (Table 3 and Table 4), although the salt concentration used in our study had a greater effect on individual fitness (Table 1). Consequently, changes in heritability were more complex than a simple positive relationship between stress effects and fitness reduction. The heritability decrease in polluted environments may also depend on how interference disrupts gene expression [3]. However this needs to be investigated by studies that focus on heritability in various polluted environments at different pollutant concentration ranges.

## Conclusion

The use of *C*. *elegans* lines allowed us to analyse, in one generation, the effects of pollutants on the genetic structure of life history traits. Our findings confirm that the decrease in heritability in polluted environments was probably due to the expression of different sets of genes, such as tolerance genes—*mtl* (metallothionein), or *hsp* (heat shock protein) genes, that disrupt genetic structure, especially when *C*. *elegans* is subject to sudden uranium pollution. Moreover the decrease in heritability was not linearly correlated to fitness reduction in polluted environments. The extremely low heritability in the uranium environment could prevent selection, despite the strong selection pressures on the traits. Finally, when individuals had adapted to one polluted environment, there seems to be a genetic cost to living in other polluted environments. Pollution can break down the genetic structure of life history traits in a population of *C*. *elegans* and thus affect its evolutionary potential.

## Supporting Information

S1 Fig Genetic covariance estimates for the traits when subjected to the different environments. Estimates are presented with their 95% Bayesian credibility intervals.
(DOCX)

S2 Fig Variance estimates for late growth when subjected to the different environments. Estimates are presented with their 95% Bayesian credibility intervals. Phenotypic variance (*V*_*P*_) is split into environmental (*V*_*E*_) and genetic variances (*V*_*G*_). Estimates were obtained using multivariate models of the different traits when subject to the same environment.
(DOCX)

S3 Fig Phenotypic correlation structures between the traits when subject to the different environments. Average values (mean ± sd) are shown for each of the 14 isogenic lines.
(DOCX)

S1 Table Deviance information criteria (DIC) for the multivariate generalized linear mixed-effects models. Models are presented for different traits within the same environment and for the same trait across different environments, with (Cov) or without (No Cov) the inclusion of genetic covariance between traits. When ΔDIC > 5, the model including genetic covariance is a better estimate of data.
(DOC)

S2 Table Environmental trait correlations for the different environments. Correlations are presented with their 95% Bayesian credibility intervals (in brackets). Values in bold are significant estimates.
(DOCX)
