# Correction: Pollution Breaks Down the Genetic Architecture of Life History Traits in *Caenorhabditis elegans*

**DOI:** 10.1371/journal.pone.0124585

**Published:** 2015-04-07

**Authors:** The PLOS ONE Staff

Fig 1 was inadvertently published with low visual quality. The journal apologizes for this error that was introduced during the publication process. The authors have provided a corrected version here.

**Fig 1:**
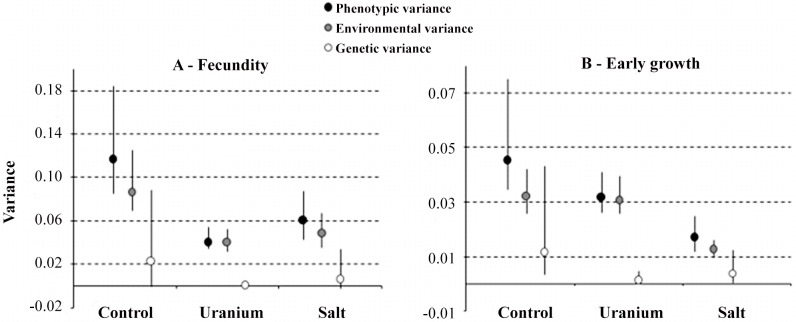
Trait variance estimates for *C*. *elegans* in the different environments. Variances are presented with their 95% intervals of Bayesian credibility. (A) fecundity and (B) early growth. Phenotypic variance (V_*P*_) is split into environmental (V_*E*_) and genetic variances (V_*G*_). Estimates were obtained using multivariate models for different traits within the same environment.
