# Supplementary material for: Pollution Breaks Down the Genetic Architecture of Life History Traits in Caenorhabditis elegans
Source: PLoS One. 2015 Feb 25;10(2):e0116214. doi: 10.1371/journal.pone.0116214 (PMC4340920; doi:10.1371/journal.pone.0116214)
Supplement: S1 Table — Models are presented for different traits within the same environment and for the same trait across different environments, with (Cov) or without (No Cov) the inclusion of genetic covariance between traits. When ΔDIC > 5, the model including genetic covariance is a better estimate of data. (DOC) [file pone.0116214.s004.doc]

**Table S1**

|  |  |  |  |  |  |
| --- | --- | --- | --- | --- | --- |
| Model | Cov | | No Cov | | ΔDIC |
| Traits in environment |  |  |  |  |  |
| Control | 25.3 |  | 41.0 |  | **15.6** |
| Uranium | -240.7 |  | -239.6 |  | 1.1 |
| Salt | -66.7 |  | -54.4 |  | **12.3** |
| Cross-environment |  |  |  |  |  |
| Fecundity | -24.0 |  | -23.4 |  | 0.6 |
| Early growth | -337.4 |  | -336.6 |  | 0.8 |
| Late growth | 311.5 |  | 312.1 |  | 0.6 |
| Survival | 488.5 |  | 488.7 |  | 0.2 |
