# Supplementary material for: Pollution Breaks Down the Genetic Architecture of Life History Traits in Caenorhabditis elegans
Source: PLoS One. 2015 Feb 25;10(2):e0116214. doi: 10.1371/journal.pone.0116214 (PMC4340920; doi:10.1371/journal.pone.0116214)
Supplement: S2 Table — Correlations are presented with their 95% Bayesian credibility intervals (in brackets). Values in bold are significant estimates. (DOCX) [file pone.0116214.s005.docx]

**Table S2**

|  |  |  |  |
| --- | --- | --- | --- |
| CONTROL | Early growth | Late growth | Survival |
| Fecundity | **0.459 [0.258, 0.630]** | 0.100 [-0.128, 0.300] | **0.737 [0.527, 0.904]** |
| Early growth |  | -0.257 [-0.417, 0.011] | 0.273 [-0.153, 0.590] |
| Late growth |  |  | 0.356 [-0.226, 0.669] |
|  |  |  |  |
| URANIUM | Early growth | Late growth | Survival |
| Fecundity | **0.670 [0.545, 0.739]** | 0.136 [-0.016, 0.334] | **0.811 [0.475, 0.905]** |
| Early growth |  | **-0.308 [-0.504, -0.182]** | **0.571 [0.114, 0.788]** |
| Late growth |  |  | 0.187 [-0.417, 0.602] |
|  |  |  |  |
| SALT | Early growth | Late growth | Survival |
| Fecundity | **0.499 [0.268, 0.623]** | **0.430 [0.297, 0.634]** | **0.431 [0.035, 0.780]** |
| Early growth |  | **0.756 [0.626, 0.804]** | **0.668 [0.217, 0.825]** |
| Late growth |  |  | **0.755 [0.536, 0.914]** |
